# Genetics of Alzheimer’s disease: an East Asian perspective

**DOI:** 10.1038/s10038-022-01050-z

**Published:** 2022-06-01

**Authors:** Akinori Miyashita, Masataka Kikuchi, Norikazu Hara, Takeshi Ikeuchi

**Affiliations:** 1 Department of Molecular Genetics, Brain Research Institute, Niigata University, Niigata, Japan; 2 Department of Genome Informatics, Graduate School of Medicine, Osaka University, Osaka, Japan; 3 Department of Computational Biology and Medical Sciences, Graduate School of Frontier Sciences, The University of Tokyo, Tokyo, Japan

**Keywords:** Alzheimer's disease, Medical genetics

## Abstract

Alzheimer’s disease (AD) is an age-related multifactorial neurodegenerative disorder. Advances in genome technology, including next generation sequencing have uncovered complex genetic effects in AD by analyzing both common and rare functional variants. Multiple lines of evidence suggest that the pathogenesis of AD is influenced by multiple genetic components rather than single genetic factor. Previous genetic studies on AD have predominantly included European ancestry cohorts; hence, the non-European population may be underrepresented, potentially leading to reduced diversity in AD genetic research. Additionally, ethnic diversity may result in dissimilar effects of genetic determinants in AD. *APOE* genotypes are a well-established genetic risk factor in AD, with the East Asian population having a higher risk of AD associated with the *APOE* ε4 allele. To date, seven genome-wide association studies (GWAS) have been conducted in East Asians, which report a total of 26 AD-associated loci. Several rare variants, including the p.H157Y variant in *TREM2*, and the p.G186R and p.R274W variants in *SHARPIN* are associated with risk of AD in East Asians. Extending genetic studies to diverse populations, including East Asians is necessary, which could yield more comprehensive insights into AD, and here we review the recent findings regarding the genetic determinants of AD from an East Asian perspective.

## Introduction

Alzheimer’s disease (AD) is an age-related neurodegenerative disorder and a leading cause of dementia. Although aging is the largest risk factor, it is not sufficient for the development of AD. The etiology of AD is complex as it involves a combination of genetic and environmental factors [1]. Studies in biological twin have estimated that the heritability of AD ranged from 58 to 79% [2]. Heritability of AD calculated based on common single nucleotide polymorphisms (SNP) was estimated to be 33% [3]. Precise knowledge of the genetic determinants of AD is essential to understand the neurobiological pathogenesis of AD.

Previous genetic studies have identified many disease-associated genes and risk variants in AD [4]. In particular, *APOE* locus is a well-established genetic risk factor for AD [5]. Genome-wide association studies (GWAS) have identified 38 different loci associated with AD [6, 7], and recent utilization of whole exome/genome sequencing (WES/WGS) and next generation sequencing (NGS) have revealed that rare coding variants not only play an important role but also have significant effects in the pathogenesis of AD [8]. Furthermore, accumulating evidence suggests that pathogenesis of AD is influenced by multiple genetic components rather than a single genetic factor [4].

Diverse genetic architectures among different ethnic groups may differentially influence how these genetic factors contribute to the pathogenesis of AD. Previous genetic studies of AD have been largely conducted in European ancestry cohorts with potential underrepresentation of non-European populations, leading to a lack of ethnic diversity in genetic research on AD. This can impede our ability to fully understand the contribution of the genetic component in the pathogenesis of AD from the viewpoint of global healthcare policy. As extending genetic studies to other populations including East Asians, could yield more comprehensive genetic insights into AD pathogenesis, this review article summarizes the recent findings on the genetic contribution to AD from an East Asian perspective.

## *APOE* Genotypes

### *APOE* as risk factor for AD

Apolipoprotein E, encoded by *APOE*, is a secreted multifunctional protein that plays central roles in lipid metabolism and the pathogenesis of neurodegenerative disorders, including AD. In the 1970s and 1980s, genetic research on *APOE* was mainly conducted from the viewpoint of dyslipidemia, and it was in the 1990s that it was reported that *APOE* genotypes confer major risk of AD [9]. Since then, genetic risk of AD associated with *APOE* ε4 and the protective role of ε2 have been confirmed worldwide [5]. *APOE* is now recognized as the strongest susceptibility gene for late-onset sporadic AD. This should be taken into account when evaluating clinical and pathological features of AD.

Three kinds of *APOE* alleles including ε2 (rs429358-rs7412, T-T [Cys-Cys]), ε3 (rs429358-rs7412, T-C [Cys-Arg]), and ε4 (rs429358-rs7412, C-C [Arg-Arg]), have been extensively evaluated as determinants of disease susceptibility. The *APOE* ε4 allele is associated with an approximately 4-fold higher risk in clinically diagnosed subjects, and this risk rises to 6-fold in patients with neuropathological confirmation [10]. Notably, the presence of such a susceptibility gene with relatively large effect size appears to be a rare phenomenon in common diseases with a sporadic occurrence. Meta-analyses of the effects of *APOE* genotypes on AD have been reported in Caucasians [11–13], Chinese [14], Indians [15], and Iranians [16]. However, there have been no report of such meta-analyses in the Japanese population.

### Larger effects of *APOE* ε4 in East Asians

An interaction between ethnicity and the effect of *APOE* genotype on AD risk has gained much attention. Specifically, while the effect of *APOE* ε4 is weaker in African American and Hispanic populations, its effect is higher in East Asian populations, including the Japanese (Table 1) [11]. Variable effects of the ε4 allele across populations can be partly explained by differences in the frequency of the ε4 allele in general population of each ethnic group. We have previously reported that the odds ratio for AD with the ε4 allele is higher in East Asians than in Europeans [17]. The frequency of the rs405509 genotypes in the promoter region of *APOE* are different between East Asian and European populations with the frequency of the T/T genotype being significantly higher in East Asians. Functional experiments using a reporter assay have demonstrated that the T genotype at rs405509 resulted in lower expression of *APOE* [17]. Thus, the modifying effect of rs405509 may explain the ethnic variability in the effects of the *APOE* ε4 allele.

**Table 1:** Genetic risk and protective effects of APOE genotypes on AD in different population

Population	Genotype: OR (95% CI)						Reference
	ε2*2	ε2*3	ε2*4	ε3*3	ε3*4	ε4*4	
Japanese	1.1 (0.1–17.2)	0.9 (0.4–2.5)	2.4 (0.4–15.4)	1.0 (Ref)	5.6 (3.9–8.0)	33.1 (13.6–80.5)	[11]
Japanese	NA	0.7 (0.3–1.6)	NA	1.0 (Ref)	3.9 (1.9–8.0)	21.8 (8.6–55.3)	[13]
Caucasians: clinic/autopsy	0.6 (0.2–2.0)	0.6 (0.5–0.8)	2.6 (1.6–4.0)	1.0 (Ref)	3.2 (2.8–3.8)	14.9 (10.8–20.6)	[11]
Caucasians: clinic/autopsy	NA	0.6 (0.3–1.2	NA	1.0 (Ref)	4.3 (3.3–5.5)	15.6 (10.9–22.5)	[13]
Caucasians: autopsy	0.1 (0.1–0.4)	0.4 (0.3–0.5)	2.7 (1.7–4.4)	1.0 (Ref)	6.1 (5.–7.4)	31.2 (16.6–58.8)	[10]
Caucasians: population-based	0.9 (0.3–2.8)	0.6 (0.5–0.9)	1.2 (0.8–2.0)	1.0 (Ref)	2.7 (2.2–3.2)	12.5 (8.8–17.7)	[11]
Caucasians: population-based	NA	0.3 (0.2–0.6)	NA	1.0 (Ref)	2.8 (2.3–3.5)	11.8 (7.0–19.8)	[13]
African Americans	2.4 (0.3–22.7)	0.6 (0.4–1.7)	1.8 (0.4–8.1)	1.0 (Ref)	1.1 (0.7–1.8)	5.7 (2.3–14.1)	[11]
Hispanics	2.6 (0.2–33.3)	0.6 (0.3–1.3)	3.2 (0.9–11.6)	1.0 (Ref)	2.2 (1.3–3.4)	2.2 (0.7–6.7)	[11]

*OR* odds ratio, *CI* confidence interval, *NA* not available, *Ref* referece

### Rare missense variants of *APOE*

Recent research in *APOE* has focused on the identification of the rare missense variants (MAF < 1%) and their functional significance [18]. The Christchurch variant rs121918393 (*APOE*_Chc_: CGC > aGC, p.Arg[R]136Ser[S]) [19–21] and the Jacksonville variant rs199768005 (*APOE*_Jax_: GTG > GaG, p.Val[V]236Glu[E]) [22, 23] have been identified as protective variants against AD in Caucasians, with the *APOE*_Chc_ variant apparently reducing the effects of the pathogenic *PSEN1* variant (GAA > GcA, p.Glu[E]280Ala[A]), which is a highly penetrant and causative mutation for dominantly inherited AD [20, 21]. Individuals carrying the *PSEN1* p.E280A mutation typically develop mild cognitive impairment at a median age of 44 years (95% confidence interval [CI]: 43‒45 years) and dementia at a median age of 49 years (95% CI: 49‒50 years). Surprisingly, a woman with homozygous *APOE*_Chc_ variant and carrying the *PSEN1* p.E280A mutation did not exhibit mild cognitive impairment until her 70 s even though abundant accumulation of amyloid-β (Aβ) was seen in the brain. However, tau accumulation in the brain, which is a major component of neurofibrillary tangles, was clearly limited, and the degree of hippocampal atrophy was also mild, suggesting that *APOE*_Chc_ may exhibit an anti-tau effect.

On the other hand, *APOE*_Jac_ was found to show an anti-Aβ effect [23] as amount of Aβ and senile plaques in the brain of *APOE*_Jax_ carriers was found to be significantly lower than that of control subjects. Additionally, biochemical analysis showed that *APOE*_Jax_ variant inhibited self-aggregation of ApoE, which may in turn inhibit the accumulation of Aβ. Genetic analysis demonstrated that *APOE*_Jax_ was equally or more protective against AD than the ε2 allele [22]. Further work is warranted to elucidate the molecular networks affected by the *APOE*_Chc_ and *APOE*_Jax_ variants. Importantly, these variants are not listed in the Japanese database of the Tohoku Medical Megabank, and it is possible that these are seen only in Caucasian. Hence, additional rare variants of *APOE* in AD patients of East Asian origin must be explored.

Among the missense variants of *APOE* identified so far, those evaluated for pathogenicity in the human genome variant database ClinVar have been summarized in Table 2. Currently, 37 variations are listed, including rs429358 and rs7412, which determine the three alleles ε2, ε3, and ε4, as well as *APOE*_Chc_ (rs121918393) and *APOE*_Jax_ (rs199768005). Many of listed variants are associated with dyslipidemia and atherosclerosis, and only three are relevant to AD (Variation ID: 242765 [rs769452], 17864 [rs429358], 694585 [rs429358 - rs121918393]). Six variants are found only in East Asians including in the Japanese (rs121918392, rs587778876, rs121918397, rs267606663, rs140808909, and rs190853081); however, none of these have been described in relation to AD. As AD can be influenced by vascular disorders that may be caused by disruption of lipid metabolism, it is important to assign biological significance to missense variants of *APOE*.

**Table 2:** Missense variants of *APOE* listed at the human genomic variant database ClinVar

Variation ID	Variant type	dbSNP ID	Location^a^	Amino acid change^b^	CADD (GRCh38-v1.6)	Anotation of the variant	Related disease	
17849	SNV	rs121918392	c.61 G > A	p.Glu21Lys	20.20	Pathogenic	Hyperlipoproteinemia, type III; and atherosclerosis (*APOE ε5*)	
440842	SNV	rs201672011	c.91 G > A	p.Glu31Lys	15.87	Pathogenic	NA	
441264	Haplotype	rs201672011 - rs769455	c.[91 G > A;487 C > T]	p.Glu31Lys - p.Arg163Cys	15.87–28.40	Pathogenic	Familial hyperlipoproteinemia, type III	
17880	SNV	rs121918399	c.127 C > T	p.Arg43Cys	23.30	Likely pathogenic	Lipoprotein glomerulopathy	
242765	SNV	rs769452	c.137 T > C	p.Leu46Pro	0.72	Conflicting interpretations: benign, likely benign, uncertain significance	Alzheimer’s disease	
441268	Haplotype	rs769452 - **rs429358**	c.[137 T > C;**388** **T** > **C**]	p.Leu46Pro - **p.Cys130Arg**	0.72–**16.65**	Pathogenic/likely pathogenic	Familial hypercholesterolemia	
17871	SNV	rs28931576	c.178 A > G	p.Thr60Ala	15.46	Pathogenic	NA	
441269	Haplotype	rs11083750 - **rs429358**	c.[305 C > G;**388** **T** > **C**]	p.Pro102Arg - **p.Cys130Arg**	23.20–**16.65**	Association	NA	
441270	Haplotype	rs28931577 - rs267606662	c.[349 G > A;508 G > C]	p.Ala117Thr - p.Ala170Pro	27.00–17.41	Pathogenic	NA	
88639	SNV	rs587778876	c.364 C > A	p.Leu122Met	24.00	Not provided	Major depressive disorder	
17864	SNV	**rs429358**	**c.388** **T** > **C**	**p.Cys130Arg**	**16.65**	Conflicting interpretations: pathogenic, likely pathogenic, risk factor, drug response, uncertain significance	Alzheimer’s disease; lipoprotein glomerulopathy; and warfarin response	
694585	Haplotype	**rs429358** - rs121918393	c.[**388** = ;460 C > A526 = ]	**p.Cys130Arg** - p.Arg154Ser	**16.65**–25.30	Protective	Alzheimer’s disease (*APOE*ε*3_*Christchurch)	
440870	Haplotype	**rs429358** - rs387906567	c.[**388** **T** > **C**;478 C > T]	**p.Cys130Arg** - p.Arg160Cys	**16.65**–28.60	Pathogenic	Familial hyperlipoproteinemia, type III	
441267	Haplotype	**rs429358** - rs267606661	c.[**388** **T** > **C**;805 C > G]	**p.Cys130Arg** - p.Arg269Gly	**16.65**–23.30	Pathogenic	Familial hyperlipoproteinemia, type III	
917851	SNV	rs1969863273	c.422 A > G	p.Gln141Arg	19.85	Uncertain significance	Familial hypercholesterolemia; familial hyperlipoproteinemia, type III; and hyperlipoproteinemia	
478904	SNV	rs267606664	c.434 G > A	p.Gly145Asp	24.50	Uncertain significance	Hypercholesterolemia	
441262	Haplotype	rs267606664 - **rs7412**	c.[434 G > A;**526** **C** > **T**]	p.Gly145Asp - **p.Arg176Cys**	24.50–**24.60**	Pathogenic	Apolipoproteinemia E1	
88640	SNV	rs587778877	c.451 C > A	p.Leu151Met	20.70	Not provided	Major depressive disorder	
17874	SNV	rs28931578	c.455 G > A	p.Arg152Gln	24.90	Pathogenic	NA	
17850	SNV	rs121918393	c.460 C > A	p.Arg154Ser	25.30	Pathogenic	Familial hyperlipoproteinemia, type III	
375636	SNV	rs200703101	c.461 G > T	p.Arg154Leu	27.80	Likely pathogenic	Abnormal circulating lipid concentration	
17851	SNV	rs769455	c.487 C > T	p.Arg163Cys	28.40	Benign	Familial hyperlipoproteinemia, type III	
17865	SNV	rs121918397	c.488 G > A	p.Arg163His	22.70	Pathogenic	Familial hyperlipoproteinemia, type III	
17879	SNV	rs121918397	c.488 G > C	p.Arg163Pro	25.60	Pathogenic	Lipoprotein glomerulopathy	
17858	SNV	rs121918394	c.490 A > C	p.Lys164Gln	25.50	Pathogenic	Hyperlipoproteinemia, type III	
17857	SNV	rs121918394	c.490 A > G	p.Lys164Glu	26.10	Pathogenic	Familial hyperlipoproteinemia, type III	
1077013	SNV	NA	c.494 G > C	p.Arg165Pro	27.50	Likely pathogenic	Lipoprotein glomerulopathy	
126456	3 bp microsatellite	rs515726148	c.497TCC[1]	p.Leu167del	–	Pathogenic	Sea-blue histiocyte syndrome	
17848	SNV	**rs7412**	**c.526** **C** > **T**	**p.Arg176Cys**	**24.60**	Drug response	Hypercholesterolemia; familial hyperlipoproteinemia, type III; warfarin response; atorvastatin response efficacy	
441265	Haplotype	**rs7412** - rs267606663	c.[**526** **C** > **T**;725 G > A]	**p.Arg176Cys** - p.Arg242Gln	**24.60**–8.57	Pathogenic	Familial hyperlipoproteinemia, type III	
441266	Haplotype	**rs7412** - rs199768005	c.[**526** **C** > **T**;761 T > A]	**p.Arg176Cys** - p.Val254Glu	**24.60**–25.20	Pathogenic	Familial hyperlipoproteinemia, type III	
17862	SNV	rs121918396	c.683 G > A	p.Trp228Ter	35.00	Pathogenic	Hyperlipoproteinemia, type III (*APOE* ε*3_*Washington); and familial hyperlipoproteinemia, type III	
1315806	SNV	rs567353589	c.688 G > A	p.Glu230Lys	11.14	Uncertain significance	Lipoprotein glomerulopathy	
17859	SNV	rs121918395	c.736 C > T	p.Arg246Cys	24.20	Pathogenic	NA	
441263	Haplotype	rs140808909 - rs190853081	c.[784 G > A;787 G > A]	p.Glu262Lys - p.Glu263Lys	23.20–24.80	Pathogenic	Hyperlipoproteinemia, type III; and atherosclerosis (*APOE ε7*)	
17875	SNV	rs121918398	c.875 G > A	p.Arg292His	25.20	Pathogenic	NA	
17876	SNV	rs28931579	c.940 A > C	p.Ser314Arg	13.28	Pathogenic	NA	

SNV rs429358 and rs7412, which determine the three common APOE alleles (ε2, ε3, and ε4) are shown in bold. Genomic variants detected only in East Asians are underlined: database searched, gnomAD v2.1.1 and v3.1.2

[Web site] CADD https://cadd.gs.washington.edu; ClinVar https://www.ncbi.nlm.nih.gov/clinvar; gnomAD https://gnomad.broadinstitute.org.

*CADD* combined annotation dependent depletion, *NA* not assigned, *SNV* single nucleotide variant

^a^Position on the APOE-encoding sequence, 1–954 bp (ATG [Met] - TGA [Ter]: 317 aa).

^b^Position on the immature APOE protein consisting of 317 amino acids, including the N-terminal signal peptide region with 18 amino acids (position, 1–18)

## GWAS

### East Asian populations

**Fig. 1:**
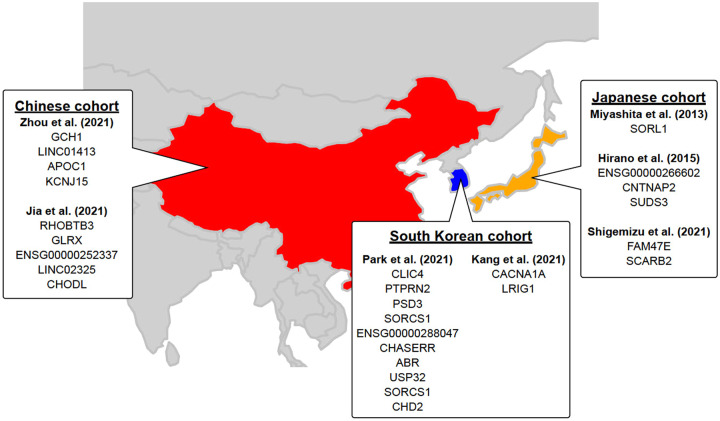
Overview of the genetic loci reported by seven GWAS in East Asian population. Note that the neighbor genes of each SNP shown below are mapped to GENCODE Release 39 (GRCh38.p13) based on rs numbers and may differ from the neighbor genes listed in the original paper

GWASs have been performed worldwide to identify common genetic factors that can explain clinical phenotypes, wherein the association between all autosomal SNPs, which are mainly genotyped by SNP arrays, and phenotypes are evaluated. The most recent GWAS for AD was performed in a European population, including 1,126,563 individuals and identified 38 susceptibility loci [7]. It is essential to perform GWASs using samples from each ethnic population to identify race-specific AD susceptibility loci. To date, 7 GWASs have been conducted in East Asians with samples from Japan, China and South Korea, and they have identified 26 AD associated loci (Fig. 1).

### Japanese cohorts

The first GWAS for AD in East Asia was reported from Japan in 2013. This study included a discovery cohort of 1008 AD patients and 1016 healthy subjects, and identified 6 SNPs outside the *APOE* region [24]. Among these, SNP rs4598682 in *SORL1* was confirmed in a replication cohort that included 885 AD patients and 985 healthy controls. Importantly, SNPs in *SORL1* have also been identified as susceptibility loci in European populations [7, 25], and in a transethnic meta-analysis that included South Korean and Caucasian cohorts.

The second GWAS for AD in East Asians was published in 2015 [26], which was a meta-analysis of a discovery cohort (816 AD patients and 7992 healthy subjects) and a replication cohort (1011 AD patients and 7212 healthy subjects). This GWAS identified rs1992269 located at 18p11.32, and meta-analysis after stratification of the discovery and replication cohorts by *APOE* ε4 carrier and non-carrier status identified rs802571 in the intron of *CNTNAP2* and rs11613092 in the intergenic region between *SUDS3* and *SRRM4*. However, a meta-analysis of *APOE* ε4 carriers did not yield any significant SNPs associated with AD.

Shigemizu et al. investigated a discovery cohort of 8036 individuals, including approximately 2000 individuals who had participated in a previous study [24, 27]. They identified 134 markers located in nine genes that satisfied the significance level in the discovery cohort, and their evaluation in the replication cohort revealed the presence of rs920608 on *FAM47E* and *SCARB2*.

### Chinese cohorts

Two GWASs have been conducted in the Chinese population since 2018. Zhou et al. obtained WGS data from 477 AD patients and 2187 healthy subjects [28], and association analysis, which excluded the *APOE* region, identified four SNPs located in *GCH1*, *APOC1*, *KCNJ15*, and *LINC01413*. Additionally, a transethnic meta-analysis of three European cohorts (ADNI, ADC, and LOAD) also identified rs72713460, which was located 11.7 kb downstream from *GCH1* and rs928771, located in the intron of *KCNJ15*. Jia et al. analyzed 1595 AD patients and 2474 healthy subjects, and identified 34 candidate SNPs [29], that were validated in a replication cohort of 2234 AD patients and 7319 healthy subjects. Four novel SNPs were present in the 34 candidate SNPs, and among these novel SNPs, rs3777215 was located in the intron regions of *RHOBTB3* and *GLRX*, while rs6859823 was located in the intergenic region of *ENSG00000251574* and *ENSG00000252337*, both of which are RNA genes. Further, rs234434 was located between RNA gene *ENSG00000285584* and noncoding RNA *LINC02325*, and rs2255835 was located in the intron region of *CHODL*.

### South Korean cohorts

Two GWAS have been recently reported from South Korea. Park et al. focused on *APOE* ε4 carriers and individuals regardless of ε4 status [30]. In the GWAS focusing on *APOE* ε4 carriers, a discovery cohort including 331 AD patients and 169 healthy subjects and a replication cohort of 190 AD patients and 97 healthy subjects, whose samples were analyzed by WGS and a custom array. Two SNPs were identified in this analysis: rs1890078, located 54 kb upstream of *SORCS1*, and rs12594991, located in the intron of *CHD2*. The authors also analyzed samples from 874 AD patients and 1063 healthy subjects, including the *APOE* ε4 carriers described above, and identified nine suggestive variants. These included two SNPs located around *SORCS1*, which were present only in ε4 carriers. Kang et al. performed a GWAS using their own South Korean cohort and Japanese samples used previously [24, 31]. The discovery cohort included 1172 South Korean AD patients and 1119 South Korean healthy subjects, while the replication cohort used samples from 976 Japanese AD patients and 980 Japanese healthy subjects. At a significance level of *P* < 5 × 1e-5, only *APOE* regions were associated in both cohorts. Next, a stratified analysis of *APOE* ε4 carriers and noncarriers yielded no significant SNPs in ε4 carriers, but rs189753894, located upstream of 7 kb from *CACNA1A*, and rs2280575, present in the intron of *LRIG1*, were found in ε4 noncarriers. Interestingly, these two SNPs had the same directionality of effect in both South Korean and Japanese cohorts and satisfied a significance level of *P* < 5e-8 during a meta-analysis.

Intriguingly, no significant SNPs were found in *APOE* ε4 carriers in two GWAS populations [26, 31], suggesting that *APOE* genotypes in ε4 carriers may account for almost all genetic determinants in AD. In contrast, several SNPs been identified in ε4 noncarriers, but they were not common, and they had a much smaller effect size than SNPs in the *APOE* region, suggesting that polygenic effects may play a role in the pathogenesis of AD in ε4 noncarriers.

### East Asian specific loci

**Fig. 2:**
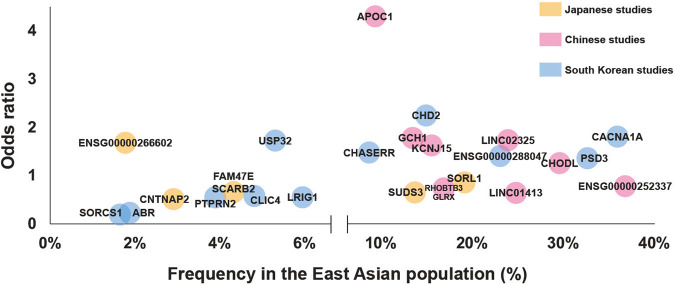
Effects and frequencies of the AD susceptible loci in East Asian population

We have summarized the statistics for AD-susceptibility loci found in the three countries in Table 3 and Fig. 2, and show large differences in frequency between East Asian and European populations for some variants. For example, rs189753894 near *CACNA1A*, found in *APOE* ε4 noncarriers in the South Korean population, had an MAF of 0.3598 in East Asian populations, while the MAF in the European population was 0.02503. On the other hand, rs12594991, which is located in the intron of *CHD2* and was found in another South Korean cohort, was less frequent in East Asians compared to Europeans. Thus, these observations explain ethnicity specific AD-susceptibility loci in East Asians.

**Table 3:** Statistics of AD susceptibility loci found in Japanese, Chinese and South Korean populations

SNP^a^	CHR:POS (GRCh38)^b^	Nearest gene^c^	Location	The number of subjects in East Asian^d^ (Discovery cohort / Replication cohort)	Healthy controls	Stratification	OR^e^	MAF (gnomAD v3.1.2)^f^			Genotyping platform	Reference
				AD patients				East Asian	non-Finnish European	MAF difference^g^		
Japanese studies												
rs4598682	11:121,505,242	*SORL1*	intron	891/885	844/985	All samples	0.75	0.192	0.01884	0.173	SNP array, PCR assay	[24]
rs1992269	18:1,872,316	*ENSG00000266602*	intron	816/1011	7992/7212	All samples	1.66	0.01777	0.1819	−0.164	SNP array, PCR assay	[26]
rs802571	7:146,265,094	*CNTNAP2*	intron	489/528	6463/5824	APOEε4 non-carrier	0.52	0.02928	0.03317	−0.004	SNP array, PCR assay	[26]
rs11613092	12:118,455,443	*SUDS3*	intergenic	323/480	1484/1364	APOEε4 carrier	0.61	0.1367	0.06899	0.068	SNP array, PCR assay	[26]
rs920608	4:76,217,307	*FAM47E, SCARB2*	intron	3962/1216	4074/2446	All samples	0.65	0.0433	0.009	0.034	SNP array, PCR assay	[27]
Chinese studies												
rs72713460	14:54,830,325	*GCH1*	intergenic	477	2187	All samples	1.74	0.1339	0.2188	−0.085	WGS	[28]
rs2591054	15:57,320,212	*LINC01413*	intron	477	2187	All samples	0.61	0.2489	0.4126	−0.164	WGS	[28]
rs73052335	19:44,916,825	*APOC1*	intron	477	2187	All samples	4.27	0.09286	0.1247	−0.032	WGS	[28]
rs928771	21:38,291,838	*KCNJ15*	intron	477	2187	All samples	1.59	0.1549	0.4975	−0.343	WGS	[28]
rs3777215	5:95,786,296	*RHOBTB3, GLRX*	intron	1595/2234	2474/5085	All samples	0.69	0.1683	0.2307	−0.062	SNP array, MALDI-TOFMS	[29]
rs6859823	5:106,218,683	*ENSG00000252337*	intergenic	1595/2234	2474/5085	All samples	0.74	0.3688	0.425	−0.056	SNP array, MALDI-TOFMS	[29]
rs234434	14:97,354,683	*LINC02325*	intergenic	1595/2234	2474/5085	All samples	1.71	0.2398	0.3077	−0.068	SNP array, MALDI-TOFMS	[29]
rs2255835	21:18,119,346	*CHODL*	intron	1595/2234	2474/5085	All samples	1.23	0.296	0.6536	−0.358	SNP array, MALDI-TOFMS	[29]
South Korean studies												
rs12063304	1:24,745,177	*CLIC4*	intergenic	543/331	169/894	All samples	0.57	0.04822	0.03179	0.016	WGS, SNP array	[30]
rs80020083	7:158,491,056	*PTPRN2*	intron	543/331	169/894	All samples	0.56	0.03888	0.0003527	0.039	WGS, SNP array	[30]
rs967326	8:18,689,751	*PSD3*	intron	543/331	169/894	All samples	1.33	0.3275	0.0523	0.275	WGS, SNP array	[30]
rs144835823	10:107,242,334	*SORCS1*	intergenic	543/331	169/894	All samples	0.32	0.01719	0.0000294	0.017	WGS, SNP array	[30]
rs78442236	10:107,273,420	*SORCS1*	intergenic	543/331	169/894	All samples	0.21	0.01676	0.0000294	0.017	WGS, SNP array	[30]
rs74352072	11:119,921,300	*ENSG00000288047*	intergenic	543/331	169/894	All samples	1.39	0.2301	0.0004703	0.230	WGS, SNP array	[30]
rs79919241	15:92,891,403	*CHASERR*	intron	543/331	169/894	All samples	1.45	0.08674	0.046	0.041	WGS, SNP array	[30]
rs201351606	17:1,179,646	*ABR*	intron	543/331	169/894	All samples	0.23	0.01897	0.00004415	0.019	WGS, SNP array	[30]
rs117665140	17:60,203,564	*USP32*	intron	543/331	169/894	All samples	1.74	0.05327	0.00001471	0.053	WGS, SNP array	[30]
rs1890078	10:107,218,478	*SORCS1*	intergenic	331/190	169/97	APOEε4 carrier	0.43	0.0792	0.0689	0.010	WGS, SNP array	[30]
rs12594991	15:92,973,197	*CHD2*	intron	331/190	169/97	APOEε4 carrier	2.21	0.1496	0.5297	−0.380	WGS, SNP array	[30]
rs189753894	19:13,513,675	*CACNA1A*	intron	976/815	621/435	APOEε4 non-carrier	1.787	0.3598	0.02503	0.335	SNP array	[31]
rs2280575	3:66,492,439	*LRIG1*	intron	976/815	621/435	APOEε4 non-carrier	0.544	0.05939	0.2958	−0.236	SNP array	[31]

*SNP* single nucleotide polymorphism, *CHR* chromosome, *POS* genomic postion, *MAF* minor allele frequency, *OR* odds ratios calculated according to the minor allele

^a^Each SNP is a representative SNP identified from the East Asian cohort

^b^Genomic position was based on GRCh38

^c^The nearest genes were based on GENCODE V39

^d^The number of subjects shows QC-passed East Asian subjects used in each study

^e^Odds ratio indicates a value calculated in the final East Asian cohort, not including non-East Asian cohort(s)

^f^MAF was provided by gnomAD v3.1.2.

^g^MAF difference was calculated by subtracting MAF in East Asian from that in non-Finnish European

Notably, none of the GWASs mentioned above identified the same loci, excluding the *APOE* region, even in the same country. One reason for these inconsistent results may be differences in the genetic background among East Asian populations. Although Japanese, Chinese, and South Koreans share genetic extensions, genetic clusters in each population are clearly distinct [32]. Even within the same country, there are several subpopulations with slightly different genetic backgrounds [33, 34]. Furthermore, there are concerns that because these GWAS are relatively smaller compared to the large GWAS in Caucasians, there may be insufficient statistical power. Thus, in the future, integrated analysis of multiple cohorts from multiple neighboring countries can help to resolve these limitations.

### Rare variants

The advent of NGS has facilitated genetic analysis at the resolution of a single nucleotide, thereby shifting the focus from common variants to the identification of rare variants. Much attention has been paid to low-frequency functional variants involving amino acid alterations, because functional rare variants may be directly linked to disease pathogenesis due to their biological consequences. Thus, rare variants with functional relevance are likely to provide a better understanding of disease etiology than common variants with small effect size that are located in the noncoding regions and are the focus of GWAS. Indeed, many rare functional variants have been successfully identified in AD in recent years, which have shed new light on the pathogenesis of AD.

The first well-known rare variant for AD is the p.R47H variant (rs75932628) in *TREM2*, which was independently identified by two research groups in 2013 [35, 36]. Since then, multiple studies have attempted to validate its genetic association with AD, and a recent GWAS of nearly 100,000 individuals has estimated an odds ratio of 2.08 with a *P* value of 2.7 × 10^−15^ for this variant [37]. Although the allele frequency of p.R47H is as low as 0.8% [37], it confers a high risk for AD, which is comparable to that of *APOE* ε4. Crucially, such a large effect size is characteristic of functional rare variants, which is in contrast to common variants with a small effect size.

However, genetic studies in East Asian populations have been unable to replicate the significance of the p.R47H variant in *TREM2*, because it is rarely found in this population. To date, thousands of Chinese and Japanese have been screened for p.R47H variant, and only three Japanese carriers of this variant have been reported (Table 4) [38–42]. This observation is also true for the rare variant p.A673T (rs63750847) in *APP*, which was identified in Icelanders and was shown to have a strong protective effect against age-related cognitive decline as well as AD [43]. The p.A673T variant was observed in control subjects aged over 85 years at a frequency of 0.45%, which is higher than that seen in AD patients [43]. However, this variant has never been reported in East Asian populations (Table 3) [44, 45].

**Table 4:** Rare variants associated with AD in East Asian populations

Gene	dbSNP	Coding DNA	Protein	Minor allele frequency			Association study in East Asians			
				European (non-Finnish)	South Asian	East Asian	Ethnicity	*P* value	OR	95% CI
*APP*	rs63750847^a^	c.2017G > A	p.Ala673Thr	0.0349%	0.0000%	0.0000%	Chinese	No carriers found		
							Chinese	No carriers found		
*MLKL*	rs763812068	c.142 C > T	p.Gln48Ter	0.0008%	0.0000%	0.1003%	Chinese	0.006	NA	NA
*TREM2*	rs75932628^a^	c.140 G > A	p.Arg47His	0.2466%	0.2321%	0.0000%	Japanese	1.00	0.57	0.05–6.30
							Chinese	No carriers found		
							Chinese	No carriers found		
	rs201280312	c.389 C > T	p.Ala130Val	0.0000%	0.0000%	0.0109%	Chinese	0.13	NA	NA
	rs2234255	c.469 C > T	p.His157Tyr	0.0294%	0.0719%	0.1805%	Japanese	0.53	1.73	0.49–6.13
							Chinese	0.005	11.01	1.38–88.05
	rs200820365	c.547 A > T	p.Ser183Cys	0.0000%	0.0000%	0.8525%	Chinese	0.035	2.75	1.03–7.34
	rs150277350	c.574 G > A	p.Ala192Thr	0.0080%	0.0294%	0.0401%	Chinese	0.18	NA	NA
	rs1391283629	c.600 G > C	p.Trp200Cys	0.0000%	0.0000%	0.0056%	Chinese	1.00	NA	NA
	rs2234256	c.632 T > C	p.Leu211Pro	0.1115%	5.8400%	0.1604%	Japanese	0.30	0.71	0.39–1.28
*PLD3*	rs145999145^a^	c.694 G > A	p.Val232Met	0.5325%	0.0851%	0.0150%	Chinese	No carriers found		
*SHARPIN*	rs572750141	c.556 G > A	p.Gly186Arg	0.0018%	0.0065%	0.0558%	Japanese	0.000012	6.1	2.4–15.5
	rs77359862	c.820 C > T	p.Arg274Trp	0.0086%	0.0818%	3.7530%	Japanese	0.0016	1.43	1.15–1.78
*UNC5C*	rs137875858^a^	c.2504 C > T	p.Thr835Met	0.0496%	0.0033%	0.0000%	Chinese	No carriers found		
	NA	c.2508 C > G	p.Val836Val	NA	NA	NA	Chinese	0.36	NA	NA
	rs368284839	c.2510 C > A	p.Thr837Lys	0.0000%	0.0000%	0.1203%	Chinese	0.13	NA	NA
	rs779272234	c.2527 A > G	p.Ser843Gly	0.0008%	0.0000%	0.1203%	Chinese	0.36	NA	NA
	rs372767649	c.2580 G > C	p.Gln860His	0.0000%	0.0294%	0.1253%	Chinese	0.017	NA	NA

Minor allele frequencies were obtained from gnomAD v2.1.1

*CI* confidence interval, *NA* not available, *OR* odds ratio

^a^rare variants proven in Caucasians

Nevertheless, several other rare variants that are significantly associated with AD have been reported in East Asians. For example, *TREM2* p.H157Y (rs2234255) has been detected not only in Caucasians [35] but also in Chinese [40] and Japanese [24], and the significance of this variant has been confirmed in the Chinese population (Table 4) [40]. Moreover, two rare variants, p.G186R (rs572750141) and p.R274W (rs77359862), identified in the coding regions of *SHARPIN*, have been reported to be associated with late-onset AD in the Japanese (Table 3) [46, 47]. Similarly, the p.R274W variant in *SHARPIN* has been associated with brain atrophy in Korean patients with AD [48]. Thus, these two are examples of rare variants that are relatively frequent in East Asians (Table 4), but have yet to be verified in other ethnic groups.

Notably, these findings raise the notion that rare variants may exist in an ethnicity dependent manner, and that they seem to exhibit a mutually exclusive behavior, i.e., wherein one rare variant seen in an ethnic group may not be found in other ethnic groups. This is probably because not enough time has passed since these rare variants arose and they are yet to spread to other populations. Alternatively, rare variants might be subjected to a selection pressure that could be detrimental to human survival, making it harder for them to spread from one population to another. Hence, to explore the significance of rare variants, it would be advantageous to analyze their impact in a genetically homogeneous population. Nonetheless, further genetic research will uncover additional rare variants associated with AD among diverse populations, and such identification may pose difficulties in validating inter-racial reproducibility. It may not be surprising even if the significance of these rare variants is not replicated in another population, and it is possible that another rare variant(s) within the same gene may be found in ethnically divergent populations. Hence, it is important to evaluate pathogenicity of each of these rare variants and utilize gene-based approaches, while also taking into account other variants observed in the same gene. Crucially, due to their rarity, genetic analysis of thousands of samples will be required to confirm significant differences.

### Future directions

During the last 20 years, numerous relevant susceptibility loci, genes and pathways associated with AD have been identified, and they have provided robust clues that have helped further our understanding of the complex pathogenesis of AD. It has also become apparent that genetic diversity among the various ethnic groups can affect disease risk, treatment efficacy, and safety. An important goal of genetic research in AD is the identification of medically actionable information that can help in the management of AD patients. Polygenic risk score, which is constructed as a weighted sum of allele counts, has been used to predict the development of AD [49]. Recent work suggests that genetic contributions to AD may be oligogenic, i.e., influenced by a limited set of common genetic variants [50]. Additional research is needed to better understand the genetic mechanisms underlying AD pathogenesis among different ethnic groups, and this could be achieved by facilitating data sharing and international collaboration. These efforts will lead to testable working hypothesis for the development of therapeutics, which would ultimately accelerate the use of precision medicine in the management of AD.
